# Using 3D gastrointestinal tract in vitro models with microfold cells and mucus secreting ability to assess the hazard of copper oxide nanomaterials

**DOI:** 10.1186/s12951-019-0503-1

**Published:** 2019-05-21

**Authors:** Victor C. Ude, David M. Brown, Vicki Stone, Helinor J. Johnston

**Affiliations:** Nano Safety Research Group, School of Engineering and Physical Sciences, Institute of Biological Chemistry, Biophysics and Bioengineering, Heriot-Watt University, Edinburgh, EH14 4AS UK

**Keywords:** Caco-2 cell, Mucus, Microfold cells, TEER, Copper oxide, Nanomaterial, Interleukin-8, Translocation, Nanotoxicology

## Abstract

**Background:**

Copper oxide nanomaterials (CuO NMs) are exploited in many products including inks, cosmetics, textiles, wood preservatives and food contact materials. Their incorporation into these products may enhance oral exposure in consumer, environmental and occupational settings. Undifferentiated and differentiated monocultures of Caco-2 cells are commonly used to assess NM toxicity to the intestine in vitro. However, the integration of other cell types into Caco-2 in vitro models increases their physiological relevance. Therefore, the aim of this study is to evaluate the toxicity of CuO NMs and copper sulphate (CuSO_4_) to intestinal microfold (M) cell (Caco-2/Raji B) and mucus secreting (Caco-2/HT29-MTX) co-culture in vitro models via assessment of their impact on barrier integrity, viability and interleukin (IL)-8 secretion. The translocation of CuO NMs and CuSO_4_ across the intestinal barrier was also investigated in vitro.

**Results:**

CuO NMs and CuSO_4_ impaired the function of the intestinal barrier in the co-culture models [as indicated by a reduction in transepithelial electrical resistance (TEER) and Zonular occludens (ZO-1) staining intensity]. Cu translocation was observed in both models but was greatest in the Caco-2/Raji B co-culture. CuO NMs and CuSO_4_ stimulated an increase in IL-8 secretion, which was greatest in the Caco-2/HT29-MTX co-culture model. CuO NMs and CuSO_4_ did not stimulate a loss of cell viability, when assessed using light microscopy, nuclei counts and scanning electron microscopy. CuO NMs demonstrated a relatively similar level of toxicity to CuO_4_ in both Caco-2/Raji B and Caco-2/HT29-MTX co- culture models.

**Conclusions:**

The Caco-2/Raji B co-culture model was more sensitive to CuO NM and CuSO_4_ toxicity than the Caco-2/HT29-MTX co-culture model. However, both co-culture models were less sensitive to CuO NM and CuSO_4_ toxicity than simple monocultures of undifferentiated and differentiated Caco-2 cells, which are more routinely used to investigate NM toxicity to the intestine. Obtained data can therefore feed into the design of future studies which assess the toxicity of substances (e.g. NMs) and pathogens to the intestine (e.g. by informing model and endpoint selection). However, more testing with a wider panel of NMs would be beneficial in order to help select which in vitro models and endpoints to prioritise when screening the safety of ingested NMs. Comparisons with in vivo findings will also be essential to identify the most suitable in vitro model to screen the safety of ingested NMs.

**Electronic supplementary material:**

The online version of this article (10.1186/s12951-019-0503-1) contains supplementary material, which is available to authorized users.

## Background

Copper oxide nanomaterials (CuO NMs) are exploited in an array of products including textiles, inks, food contact materials, intrauterine devices and wood preservatives [1, 2] primarily due to their antimicrobial properties [3]. CuO NMs are also incorporated into heat transfer fluids and/or semiconductors [4, 5]. These applications of CuO NMs may lead to their ingestion in occupational, environmental and consumer settings. For example, CuO NMs may be exposed to the gastrointestinal (GI) tract intentionally via ingestion of foods containing NMs. Accidental ingestion of CuO NMs may occur via the ingestion of food or water contaminated by leachates from food contact materials, hand to mouth contact in an occupational setting and following inhalation, due to mucociliary clearance. Although there are plausible scenarios for CuO NM exposure via the GI tract, there are a lack of studies which have assessed the toxicity of ingested NMs, including CuO NMs [6–8]. The Caco-2 cell line was first isolated from a human colon adenocarcinoma by Fogh and colleagues [9]. Monocultures of undifferentiated and differentiated Caco-2 cells have been used to study the toxicity of NMs such as zinc oxide (ZnO), TiO_2_ and CuO NMs [10–13]. However, the use of monocultures of cells may not adequately represent intestinal physiology in vivo. Incorporation of microfold (M) cells and mucus secreting cells into Caco-2 cell cultures, can increase the physiological relevance of the intestinal in vitro models.

M cells represent ~ 10% of the follicle-associated epithelium in the intestine and are responsible for antigen sampling from the GI tract lumen [7, 14–16]. M cells lack microvilli on the apical side and are responsible for transporting particles and pathogens across the intestinal epithelium to underlying immune cells [16–20]. There are currently three different in vitro models of M cells and all are based on differentiated Caco-2 co-cultures. The first is a co-culture of murine lymphocytes (isolated from Peyer’s patches) and Caco-2 cells [21]. This model has been used to study the adhesion, internalization and translocation of *Vibrio cholerae* [21] and to investigate NM transport across the intestinal epithelium [22]. The second model is a co-culture of human Burkitt’s Raji B cells with Caco-2 cells [23]. This model has been used previously to assess translocation of *E. coli*, *S. enterica* [24, 25], TiO_2_ NMs [26, 27], aminated and carboxylated polystyrene NMs [17, 23], chitosan-DNA NMs [22], and polystyrene NMs [28]. The third model involves a co-culture of Caco-2 cells with Raji B cells, but the insert of the transwell plate is inverted during the culture [17]. Translocation and impacts of Ag NMs on this model has been investigated via assessment of whole genome gene expression [29]. M cell development using inverted and un-inverted transmembrane inserts have been used to study insulin translocation across the intestinal barrier [30]. Antunes et al. reported that there was no difference between inverted and un-inverted Caco-2/Raji B co-culture based on Wheat Germ Agglutinin (WGA) staining and insulin translocation studies [30], hence the un-inverted model was selected for this study. Several methods have been used previously to confirm M cell development within in vitro models including; histology (e.g. WGA staining of sialic acid and *N*-acetylglucosamine residues present in M cells and immunostaining of M cell specific proteins), TEER measurement (which declines when M cells are present when compared to monocultures of differentiated Caco-2 cells), alkaline phosphatase secretion (which decreases when M cells are present), assessment of particle/pathogen transport (which is enhanced when M cells are present) and visualisation of cell morphology using and electron microscopy (to identify a change in the organisation of microvilli on the M cell apical surface) e.g. [17, 24, 26, 29–32, 48].

Mucus lubricates the gastrointestinal tract and helps to improve the movement of food substances and other materials, thereby enhancing digestion and absorption of food including egestion of undigested food, microorganisms and microbial by-products [33]. Mucus prevents infection and activation of inflammation, which could be damaging to the intestine by clearing and separating toxic substances and pathogens from the epithelial cells to protect the intestinal epithelium [34]. When transformed with methotrexate (MTX), the human colon adenocarcinoma cell line (HT29) can be used as a mucus secreting cell line. A monoculture of HT29-MTX cells does not sufficiently mimic the human intestinal epithelium, hence a co-culture of Caco-2 and HT29-MTX cells is used to improve physiological relevance [7]. When co-cultured with differentiated Caco-2 cells a mucus layer forms within 3 to 4 weeks, as muciporous goblet cells form [35]. The mucus layer is firmly bound, 2–10 µm thick, completely covers the co-culture surface and can withstand cell washing [36]. Culturing of Caco-2 and HT29-MTX cells at a ratio of 9:1 has been demonstrated to develop an in vitro model with mucus secretion properties resembling that of the human intestine in vivo [37, 38]. The Caco-2/HT29-MTX in vitro model has been used to investigate the toxicity of 20 and 200 nm Ag NMs and AgNO_3_ [38] as well as impact of the presence of mucus on intestinal transport of substances (such as lipophilic drugs, PEGylated solid lipid NMs, Ag NMs) [38–42].

Although the Caco-2/Raji B and Caco-2/HT29-MTX co-culture models have been used to investigate NM transport and toxicity previously [27, 30, 38, 43–46], only limited NMs (Ag, TiO_2_, polystyrene and chitosan-DNA NMs) have been tested and only limited endpoints (cell viability, IL-8 and ROS production) have been used to assess NM toxicity. Furthermore, comparisons between the responses of different in vitro intestinal models has not been routinely performed. In this study an extensive assessment of the toxicity of NMs to the intestine in vitro was investigated using M cell and mucus secreting Caco-2 co-culture models. A range of endpoints, including assessment of the impact on tight junction integrity (e.g. using transepithelial electrical resistance (TEER) measurement and immunostaining of a tight junction protein), cell morphology (e.g. using scanning electron microscopy (SEM) and light microscopy), and IL-8 production were investigated. CuO NM translocation was also investigated in this study. Previously, CuO NMs exposed to undifferentiated and differentiated monocultures of Caco-2 cells stimulated an increase in IL-8 production, and caused a decrease in barrier integrity [47]. Undifferentiated cells were most sensitive to CuO NM toxicity than differentiated cells, and a loss of cell viability was only observed in undifferentiated cells [47]. It was hypothesized in this study that CuO NMs and CuSO_4_ will induce toxicity to Caco-2/Raji B and Caco-2/HT29-MTX co-culture in vitro models. A greater impact on the Caco-2/Raji B co-culture is expected as M cells are responsible for antigen sampling and this model has less mucus to protect the cells which is likely to lead to a greater exposure of cells to CuO NMs and CuSO_4_.

## Results

### Verification of intact barrier formation by Caco-2/Raji B and Caco-2/HT29-MTX co-cultures

**Fig. 1:**
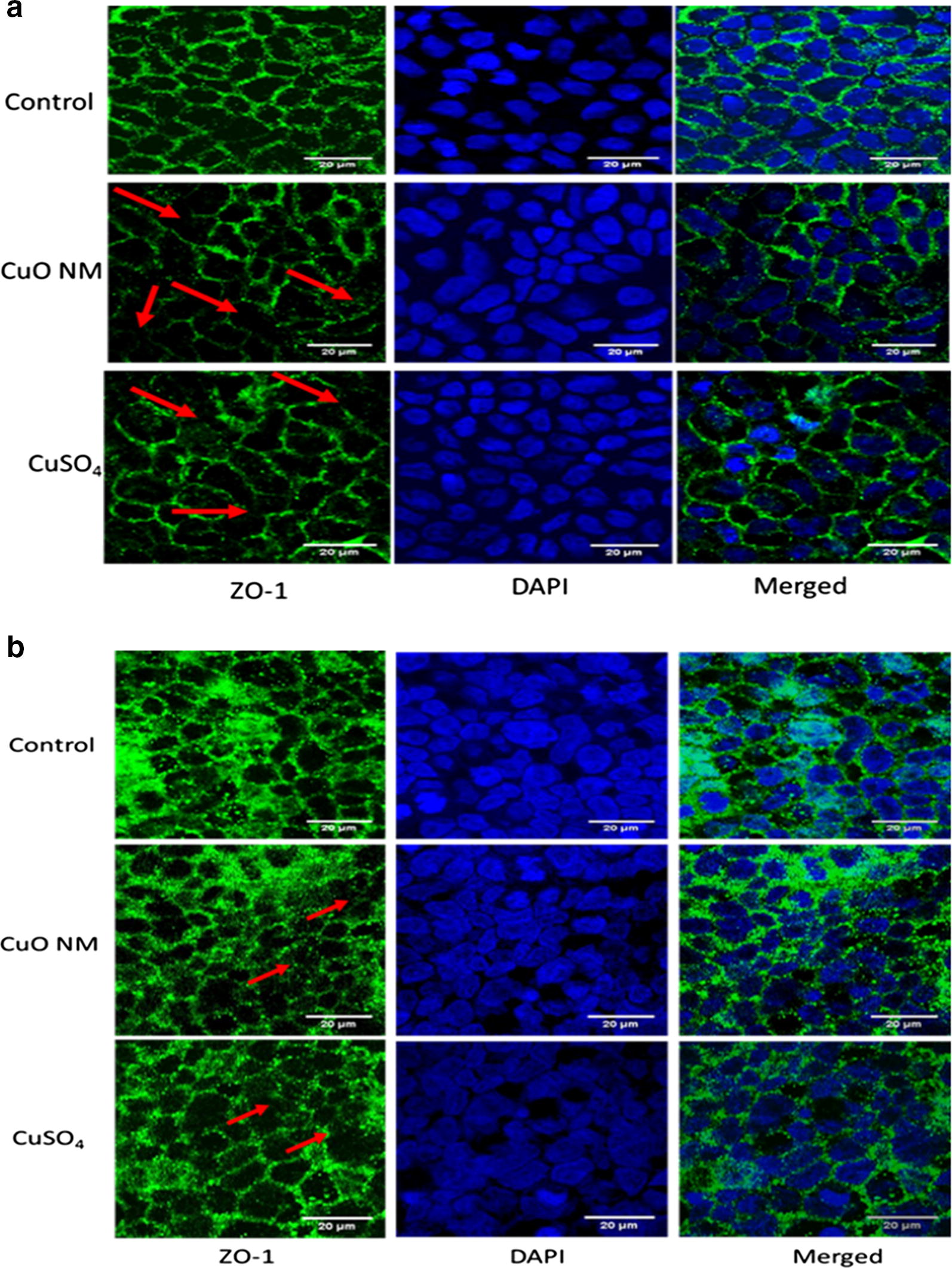
Impact of CuO NMs and CuSO_4_ on the tight junction protein ZO-1 of the co-cultures. The Caco-2/Raji B (**a**) and Caco-2/HT29-MTX (**b**) co-cultures were exposed to cell culture medium (control) or 6.34 Cu µg/cm^2^ of CuO NMs and CuSO_4_ for 24 h, then fixed, and stained for the tight junction protein ZO-1 (green) and nucleus (blue). The images of extended focus were obtained with Zeiss LSM880 confocal microscope using the Zen program for data analysis. Red arrows indicate areas where there is a reduction in ZO-1 staining intensity. Scale bar = 20 µm. Representative images are shown

The development of tight junctions in the Caco-2/HT29-MTX co-culture was monitored by TEER measurement from 5 days after seeding of the cells. The TEER value increased continuously over time from ~ 62 Ω cm^2^ on the first measurement (day 5) and reached 870 Ω cm^2^ on the 21st day (Additional file 1). The TEER values of the Caco-2/Raji B co-culture also increased continuously from first measurement on day 5 (~ 100 Ω cm^2^) to the 15th day (715 Ω cm^2^) (the day the Raji B cells were seeded to BL compartment). The TEER continued to increase until 2 days (~ 755 Ω cm^2^) after the seeding of the Raji B cells and at day 20 a decrease in TEER value to approximately 690 Ω cm^2^ was observed (Additional file 2). The presence of tight junctions (one of the characteristics of mature intestinal epithelium) in the Caco-2/Raji B and Caco-2/HT29-MTX co-culture was confirmed by staining the tight junction protein, ZO-1 (Fig. 1).

**Fig. 2:**
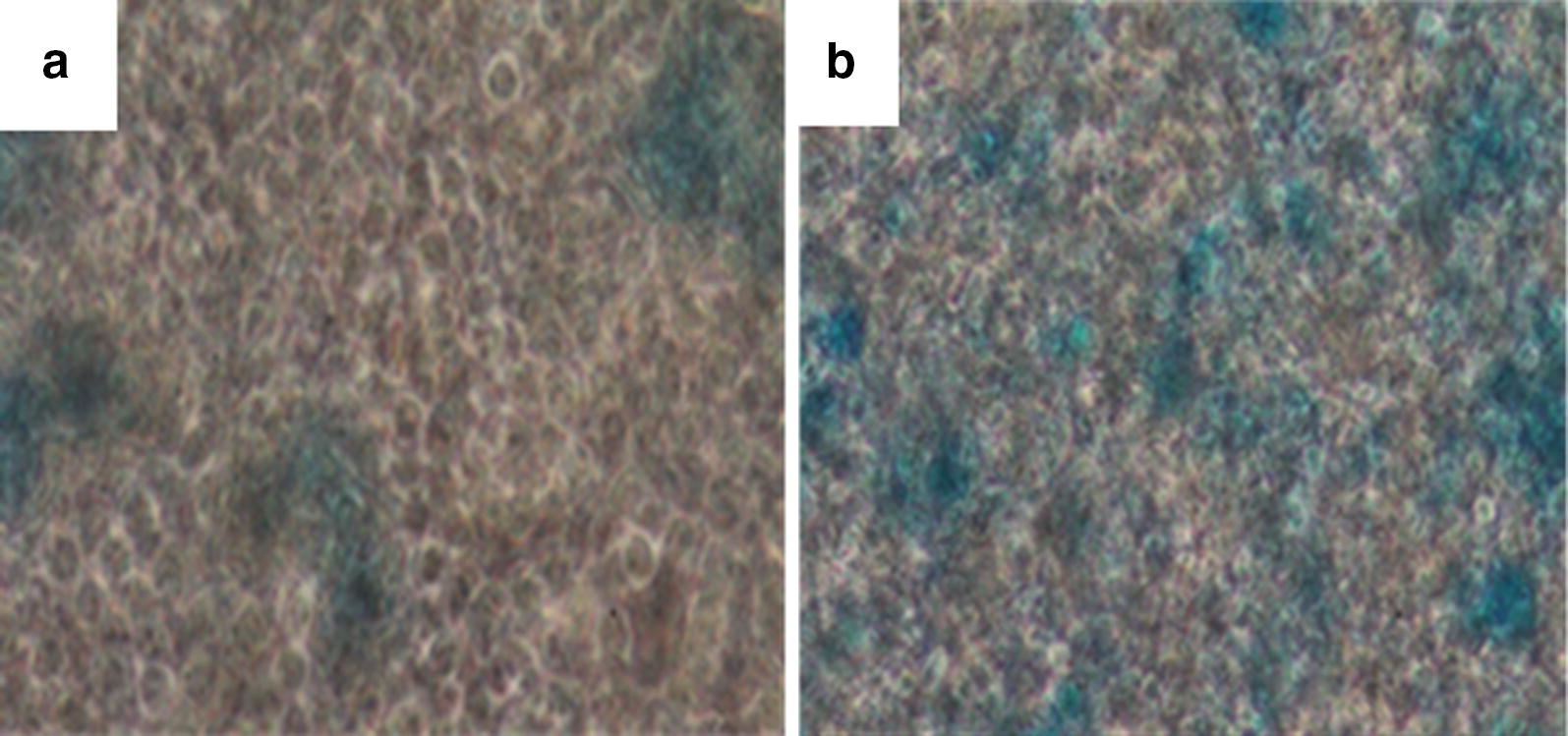
Mucus staining with Alcian Blue. The Caco-2/Raji B (**a**) and Caco-2/HT29-MTX (**b**) co-culture models were stained with Alcian blue and imaged with a ZEISS light microscope. The blue colour is mucus stained with Alcian blue. Representative images are shown

**Fig. 3:**
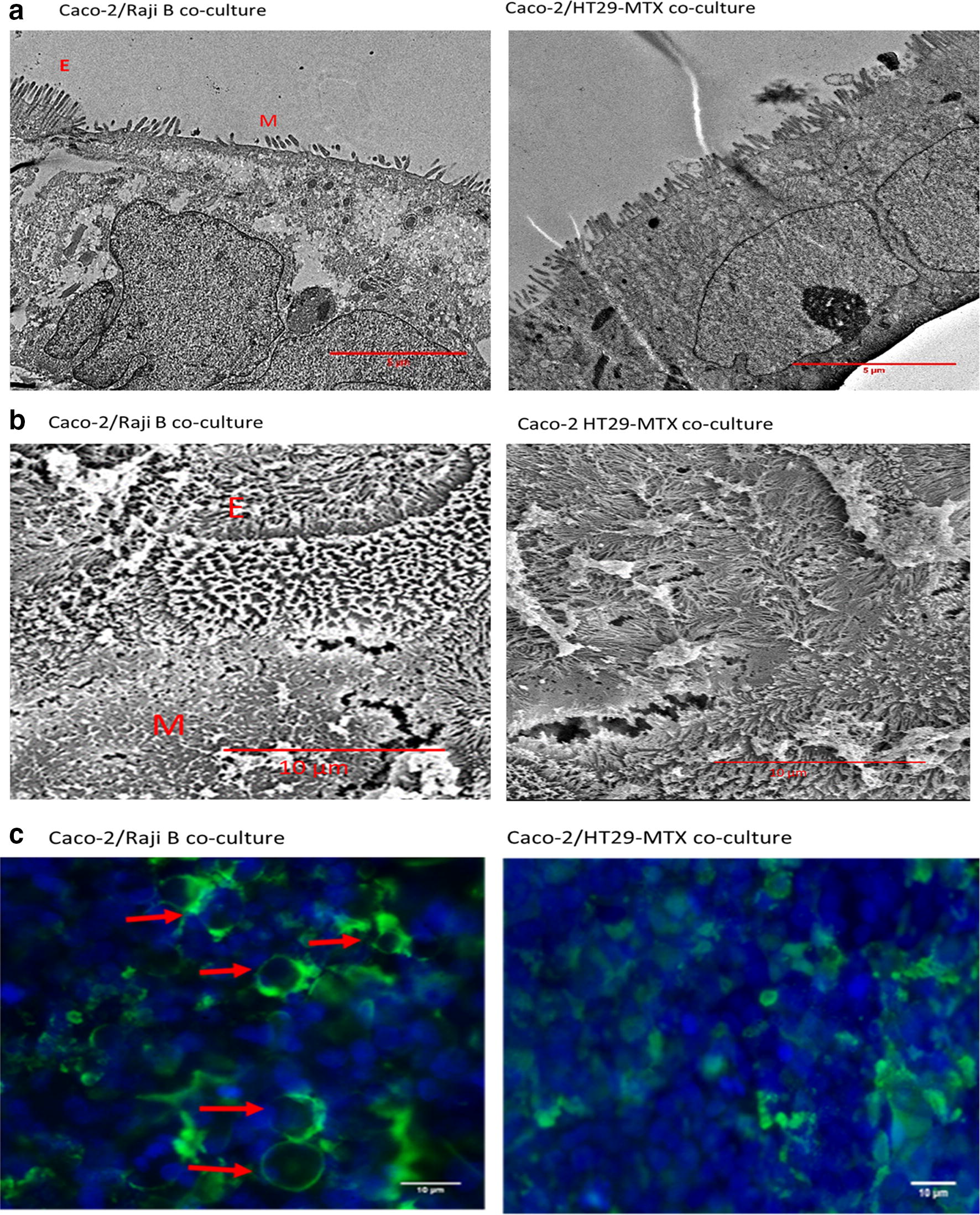
Confirmation of the presence of M cells in Caco-2/Raji B co-culture. To identify the presence of M cells Caco-2/Raji B and Caco-2/HT29-MTX co-cultures were examined by TEM (**a**) and SEM (**b**). ‘M’ indicates the presence of M cells, which have a reduced number of microvilli, and ‘E’ indicates epithelial cells with microvilli which cover the cell surface. M cells were also identified using fluorescent microscopy; Caco-2/Raji B and Caco-2/HT29-MTX co-culture models were fixed, labelled with WGA FITC (green) and mounted with ProLong with DAPI (nucleus: blue) and the images obtained with a fluorescent microscope (**b**). Red arrows indicate the presence of M cells. TEM and SEM scale bar = 5 µm, WGA staining and SEM scale bar = 10 µm. Representative images are presented

The presence of mucus on the surface or in the Caco-2/HT29-MTX co-culture was determined by staining with Alcian blue. A greater intensity of staining was observed in the Caco-2/HT29-MTX co-culture compared to the Caco-2/Raji B co-culture, suggesting that there was greater mucus production (Fig. 2). Transmission electron microscopy (TEM) and SEM were used to confirm the development of M cells by the Caco-2/Raji B co-culture (Fig. 3a, b). The TEM and SEM images demonstrated that some cells had fewer and more irregular/disorganised microvilli in the Caco-2/Raji B co-culture which suggests that this is where M cells are located (Fig. 3a, b). Microvilli were present in all the surfaces of the Caco-2/HT29-MTX co-culture (Fig. 3a, b). Furthermore, the presence of mucus on the surface of the Caco-2/HT29-MTX co-culture can be observed using SEM (Fig. 3b). WGA staining was used to identify M cells in the co-cultures. A high intensity of WGA staining was visualised in the Caco-2/Raji B co-culture, indicating the formation of M cells (Fig. 3c). Whilst some staining was observed in the Caco-2/HT29-MTX co-culture the staining was more diffuse than that observed for the Caco-2/Raji B co-culture (Fig. 3c).

### Impact of CuO NMs and CuSO_4_ on cell viability and barrier integrity

**Fig. 4:**
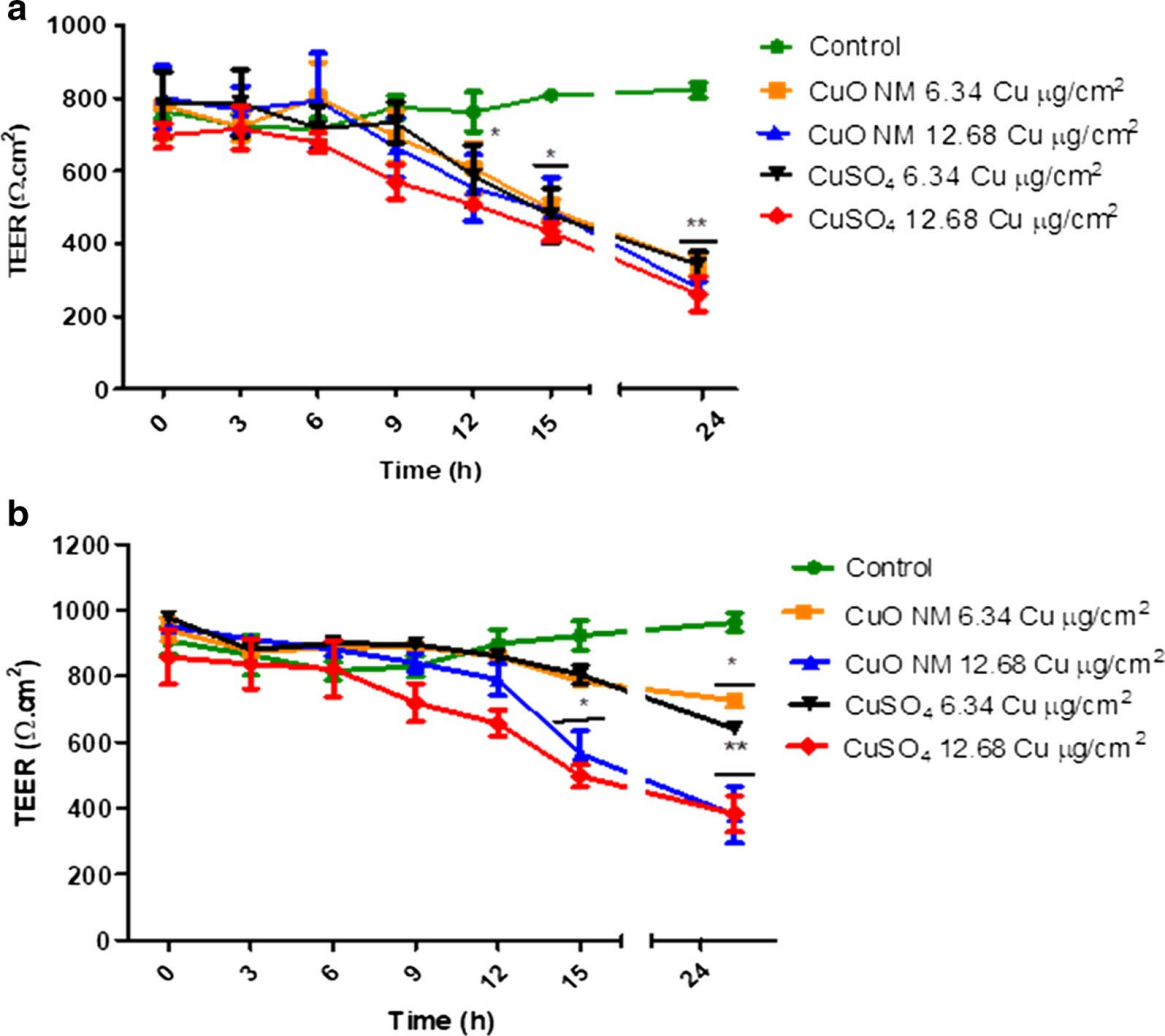
Impact of CuO NMs and CuSO_4_ on TEER values of the co-culture models. The Caco-2/Raji B (**a**) and Caco-2/HT29-MTX (**b**) co-culture models were exposed to cell culture medium (control, 0), CuO NMs or CuSO_4_ at concentrations of 6.34 or 12.68 Cu µg/cm^2^ for 24 h. TEER values were measured using epithelial volt-ohmmeter EVOM every 3 h. Data are expressed as mean TEER value ± SEM (n = 3). Significance at p < 0.05 is indicated by * and ** for p < 0.01, compared with the control

The impact of CuO NMs and CuSO_4_ on the TEER value of Caco-2/Raji B and Caco-2/HT29-MTX co-cultures was assessed as an indicator of barrier integrity (Fig. 4). The TEER value of control Caco-2/Raji B and Caco-2/HT29-MTX co-culture models was unaffected during the experiment. CuO NMs and CuSO_4_ caused a time and concentration dependent decrease in TEER values in both co-cultures (Fig. 4). A greater loss of barrier integrity was observed in the Caco-2/Raji B co-culture. The Caco-2/Raji B co-cultures demonstrated a significant time and concentration dependent decrease in TEER, with a significant effect first observed at 12 h post exposure to CuO NMs and CuSO_4_ at a concentration of 12.68 Cu µg/cm^2^ (Fig. 4a). For the Caco-2/HT29-MTX co-culture a significant decrease in TEER was first observed when exposed to 12.68 Cu µg/cm^2^ of CuO NMs and CuSO_4_ at 15 h post exposure (Fig. 4b). At 24 h, both concentrations of CuO NMs and CuSO_4_ caused a highly significant decrease in TEER in the Caco-2/Raji B co-culture (p < 0.01) and the Caco-2/HT29-MTX model [6.34 Cu  µg/cm^2^ and 12.68 Cu µg/cm^2^ (p < 0.05)].

Visualisation of the tight junction protein ZO-1 was also used to investigate impacts of CuO NMs and CuSO_4_ on barrier integrity. Following treatment with 6.34 Cu µg/cm^2^ of CuO NMs and CuSO_4_ for 24 h, the intensity of ZO-1 tight junction protein staining was similar to that observed for the control in the Caco-2/HT29-MTX co-culture. However, the Caco-2/Raji B cells appear to have a slight decrease in the intensity of the tight junction protein staining following exposure to CuO Ms and CuSO_4_ (Fig. 1).

**Fig. 5:**
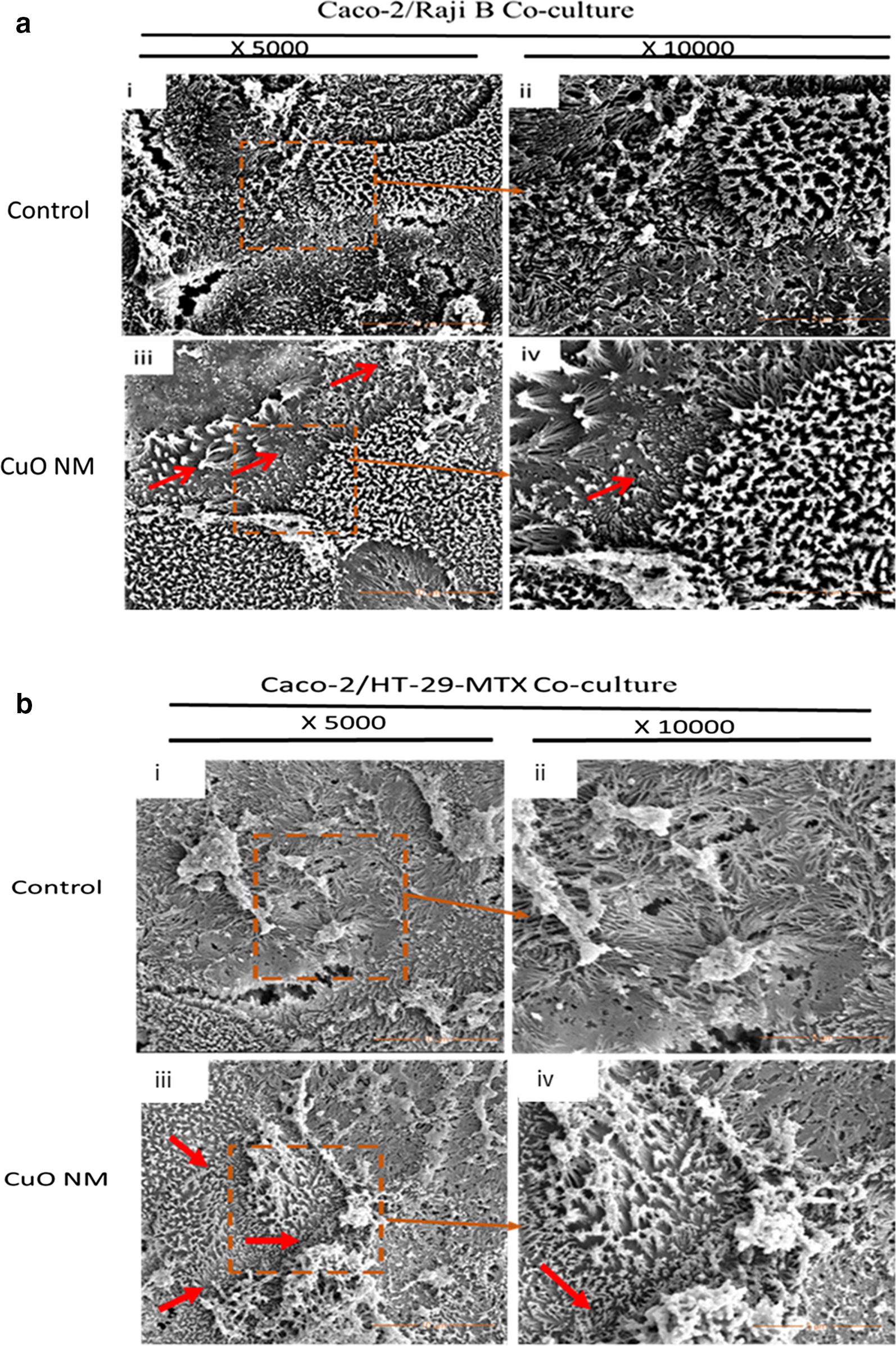
SEM imaging of the Caco-2/Raji B (**a**) Caco-2/HT29-MTX (**b**) co-culture exposed to CuO NMs for 24 h. The co-cultures were exposed to cell culture medium (control) or 12.68 Cu µg/cm^2^ of CuO NMs for 24 h. The cells were washed, fixed, dehydrated, dried and examined by SEM. Specimens **(i)** and **(ii)** are control co-cultures imaged at magnifications of ×5000 and ×10,000 respectively. **(iii)** and **(iv)** are co-cultures exposed to 12.68 Cu µg/cm^2^ of CuO NMs imaged at magnifications of ×5000 and ×10,000 respectively. Representative images are shown. Red arrows indicate area of shortened or absent microvilli

**Fig. 6:**
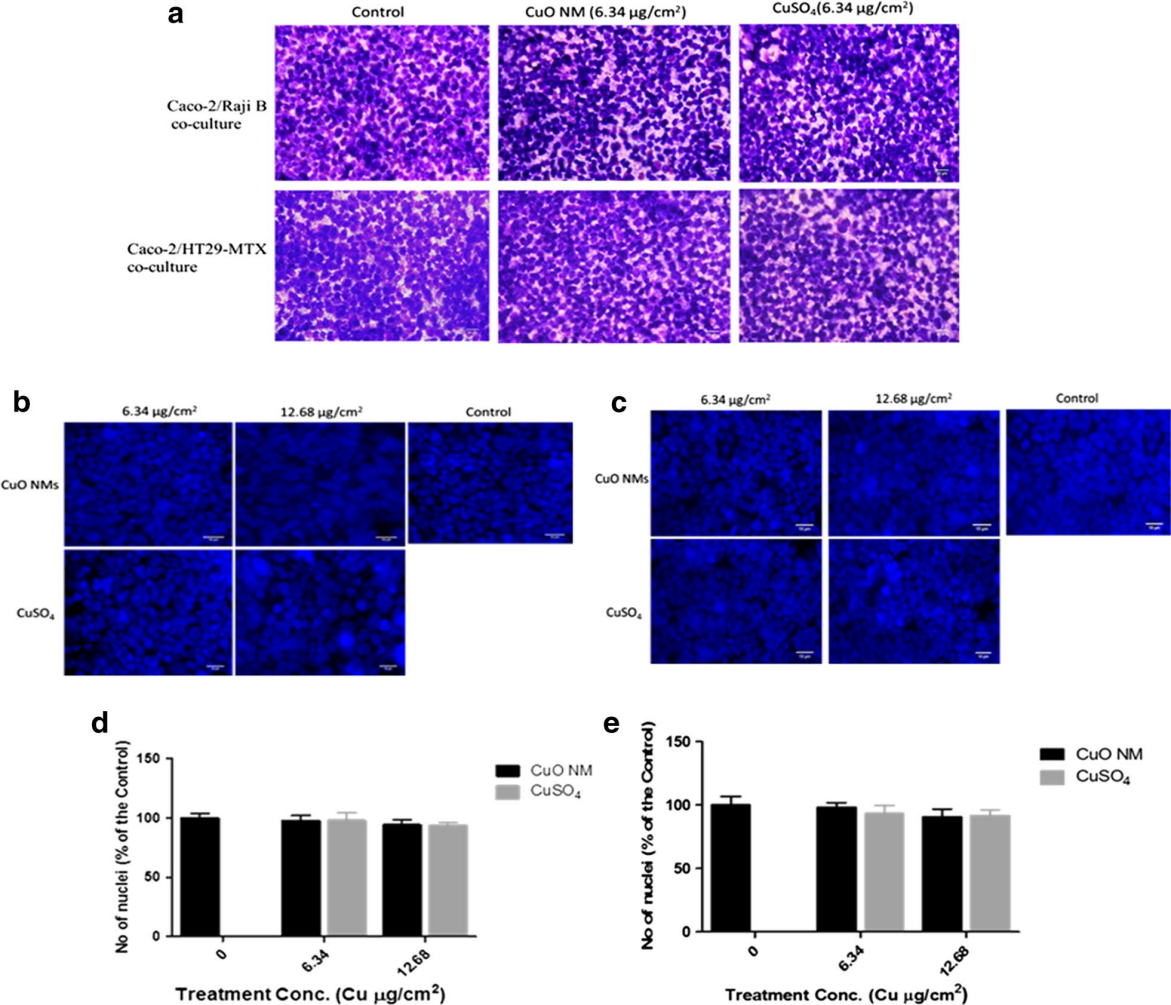
Impact of CuO NMs and CuSO_4_ on co-culture viability (cell number) and morphology. The Caco-2/Raji B and Caco-2/HT29-MTX co-cultures were exposed to cell culture medium (control, 0) or 6.34 Cu µg/cm^2^ CuO NMs or CuSO_4_ for 24 h (**a**). The cells were then fixed, stained with Rapid Romanowisky stain and visualised using light microscopy (magnification ×40, scale bar = 20 µm). To evaluate cell number Caco-2/Raji B and Caco-2/HT29-MTX co-cultures were treated with 6.34 and 12.68 Cu µg/cm^2^ of CuO NMs and CuSO_4_, fixed and the nucleus stained with DAPI (**b**, **c**). Images obtained with Zeiss fluorescent Microscope, Carl Zeiss Axio Scope A 1 Upright Research Microscope (magnification ×40, scale bar = 10 µm). Representative images are shown (n = 3). **d**, **e** Caco-2/Raji B and Caco-2/HT29-MTX co-cultures nuclei were counted using Image J software. Data are presented as the number of nuclei (expressed as % of the unexposed control) ± SEM (n = 3)

The impact of CuO NMs on cell number, cell morphology, and microvilli organisation in the Caco-2/Raji B and Caco-2/HT29-MTX co-culture models was assessed using scanning electron microscopy (SEM), fluorescent and light microscopy. Using SEM, it was evident that extended microvilli covered the entire cell surface of control Caco-2/HT29-MTX co-culture cells (Fig. 5). For the Caco-2/Raji B co-culture a reduced number of microvilli were observed, and the microvilli were more disorganised, which suggests this is where M-cells are located (Fig. 5). On exposure of both cell models to CuO NMs (12.68 Cu µg/cm^2^), the microvilli of some cells appear to have shortened, compared to control when visualised using SEM (Fig. 5). However, quantification of the length of microvilli would be required to confirm this observation. Using light microscopy, it was observed that CuO NMs and CuSO_4_ did not impact on cell morphology or cell number (Fig. 6a). For assessment of viability, the Caco-2/Raji B and Caco-2/HT29-MTX co-cultures were stained with 4, 6-diamido-2-phenylindole (DAPI) and a nuclei count performed to investigate changes in cell number. The co-cultures treated with CuO NMs or CuSO_4_ showed no significant difference in nuclei number, compared to the control (Fig. 6b, c). Representative images shown in Fig. 6b, and c confirmed this finding, as do the light microscopy images (Fig. 6a), which suggests that the treatments did not impact on cell number.

### Translocation of Cu across the intestinal monolayer

**Fig. 7:**
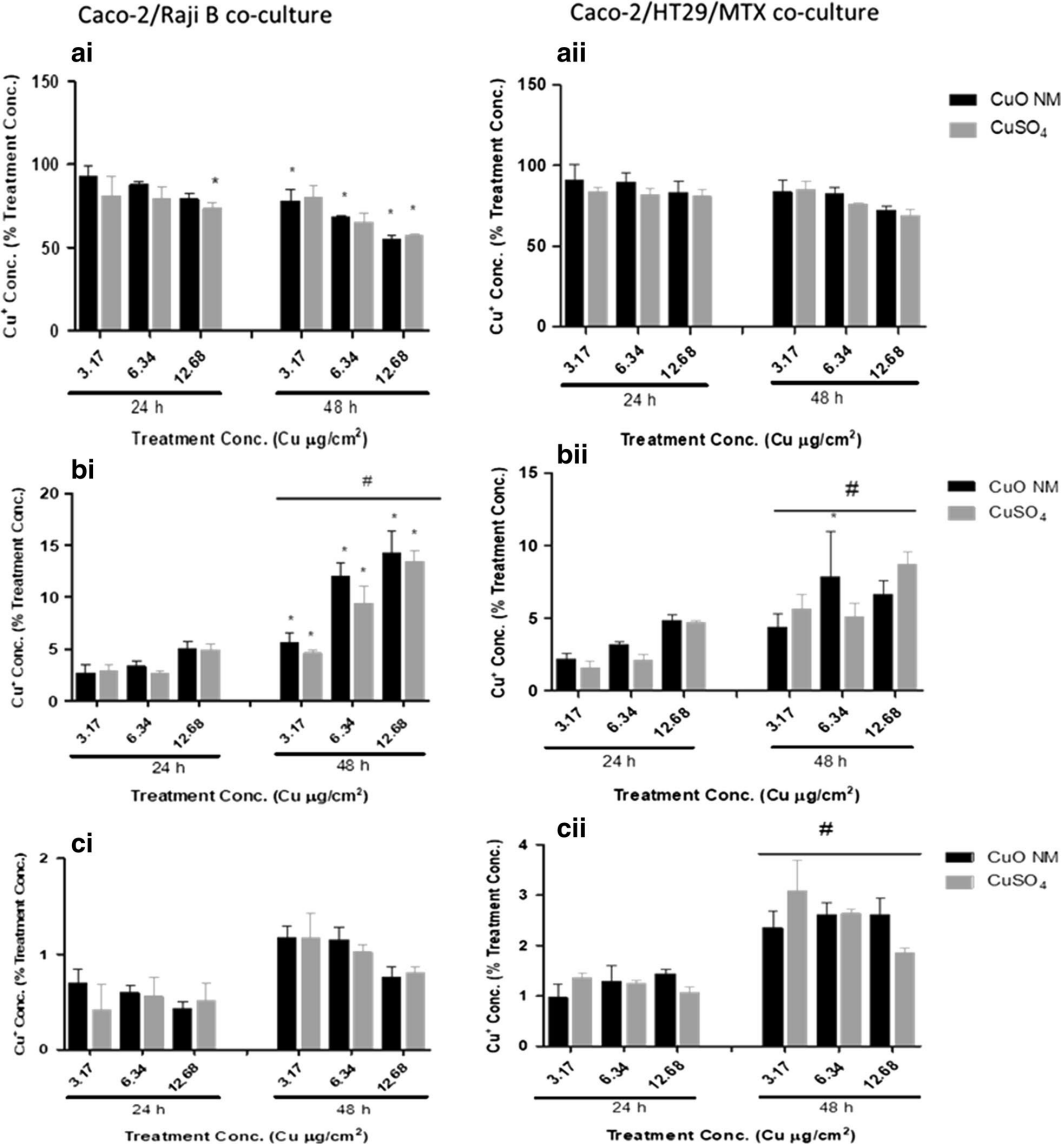
CuO NM and CuSO_4_ cellular uptake and translocation across the co-culture barrier. The Caco-2/Raji B and Caco-2/HT29-MTX co-cultures were exposed to CuO NMs or CuSO_4_ at concentrations of 3.17, 6.34 or 12.68 Cu µg/cm^2^ for 24 and 48 h. The level of Cu in the AP (**a**) and BL (**b**) compartments, and the cells (**c**) was evaluated by ICP-OES. Data are expressed as mean copper concentration (as a percentage of the exposed concentration) ± SEM (n = 3). Significance at p < 0.05 is indicted by * for comparison of CuO NMs at a concentration of 3.17 Cu µg/cm^2^ to the other treatment concentrations within each time point or # for comparison of equivalent concentrations between 24 and 48 h time points

The concentration of Cu in the apical (AP) and basloateral (BL) compartments, and the cell lysate was quantified to investigate CuO NM and CuSO_4_ translocation across the intestinal barrier in vitro 24 h post exposure of the Caco-2/Raji B co-cultures to CuO NMs and CuSO_4_. The concentration of Cu in the AP compartment ranged from 72 to 92% (expressed as percentage of the initial treatment concentration) (Fig. 7ai). At 48 h the Cu concentration in the AP compartment was between 54 and 79% (Fig. 7ai). For the Caco-2/HT29-MTX co-culture, the Cu concentration in the AP compartment after 24 h exposure to CuO NMs and CuSO_4_ ranged from 80 to 91% (of the initial exposure concentration), whereas at 48 h post exposure the Cu concentration ranged from 69 to 83 (of  % of the exposed concentration) (Fig. 7aii). Therefore, for both models the Cu concentration in the AP compartment decreased over time, with the greatest decrease observed for the Caco-2/Raji B co-culture. There was no difference between the translocation of Cu for CuO NMs and CuSO_4_ in both models.

A time dependent significant increase in the concentration of Cu in the BL compartment was observed after treatment of both co-cultures to CuO NMs and CuSO_4_, which suggests that Cu translocated from the AP compartment (Fig. 7b). However, more Cu translocated to the BL compartment in the Caco-2/Raji B co-culture compared to Caco-2/HT29-MTX co-culture. For example, Cu concentration in the BL compartment of the Caco-2/Raji B co-culture was between 2.5 and 5.2% for CuO NMs and CuSO_4_ at 24 h, and at 48 h ranged from ~ 4.6 to 14.2% (Fig. 7bi). For the Caco-2/HT29-MTX co-culture the concentration of Cu in the BL compartment at 24 h post exposure to CuO NMs and CuSO_4_ ranged from 1.6 to 4.8% and from 4.5 to 8.8% at 48 h (Fig. 7bii). The findings suggest that Cu translocation increased over time in both models, and Cu translocation was greater in the Caco-2/Raji B co-culture.

The concentration of Cu retained in cells at all concentrations and time points was less than 3.1% of the initial exposure concentration in both co-culture models (Fig. 7ci, ii). The detectable Cu in the Caco-2/Raji B co-culture cell lysate ranged between 0.4 and 1.5%, whereas the cellular retention of Cu in Caco-2/HT29-MTX co-culture ranged between 0.9 and 3.0% after exposure to CuO NMs and CuSO_4_.

**Fig. 8:**
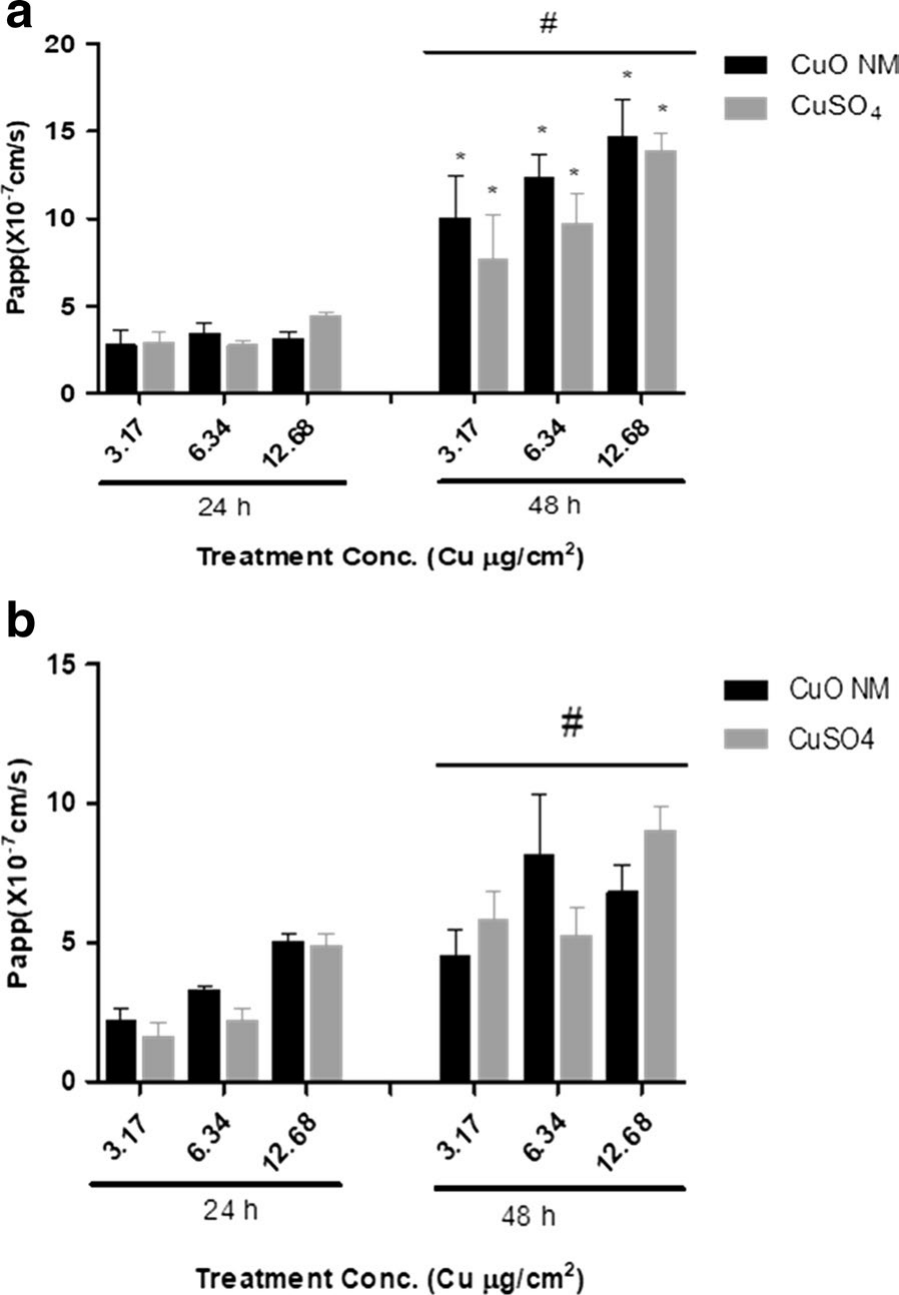
Apparent permeability coefficient (*P*_*app*_) of CuO NMs and CuSO_4_. The Caco-2/Raji B (**a**) and Caco-2/HT29-MTX (**b**) co-cultures were exposed to CuO NMs or CuSO_4_ at concentrations of 3.17, 6.34 or 12.68 Cu µg/cm^2^ for 24 and 48 h. The concentration of Cu in the AP and BL compartment was determined by ICP-OES and *P*_*app*_ was calculated. Data are expressed as mean *P*_*app*_ ×10^−7^ cm/s ± SEM (n = 3). Significance at p < 0.05 is indicted by * for comparison of CuO NMs at a concentration of 3.17 Cu µg/cm^2^ at 24 h to other treatment concentrations within each time point or # for comparison of equivalent concentrations between the 24 and 48 h time points

The apparent permeability coefficient (*P*_*app*_) of Caco-2/Raji B and Caco-2/HT29-MTX co-culture exposed to CuO NMs and CuSO_4_ showed a significant time dependent increase at all concentrations (Fig. 8), with a higher *P*_*app*_ value observed at 48 h. The Caco-2/Raji B co-culture demonstrated a higher *P*_*app*_ value indicating greater permeability compared to Caco-2/HT29-MTX co-culture.

### IL-8 production

**Fig. 9:**
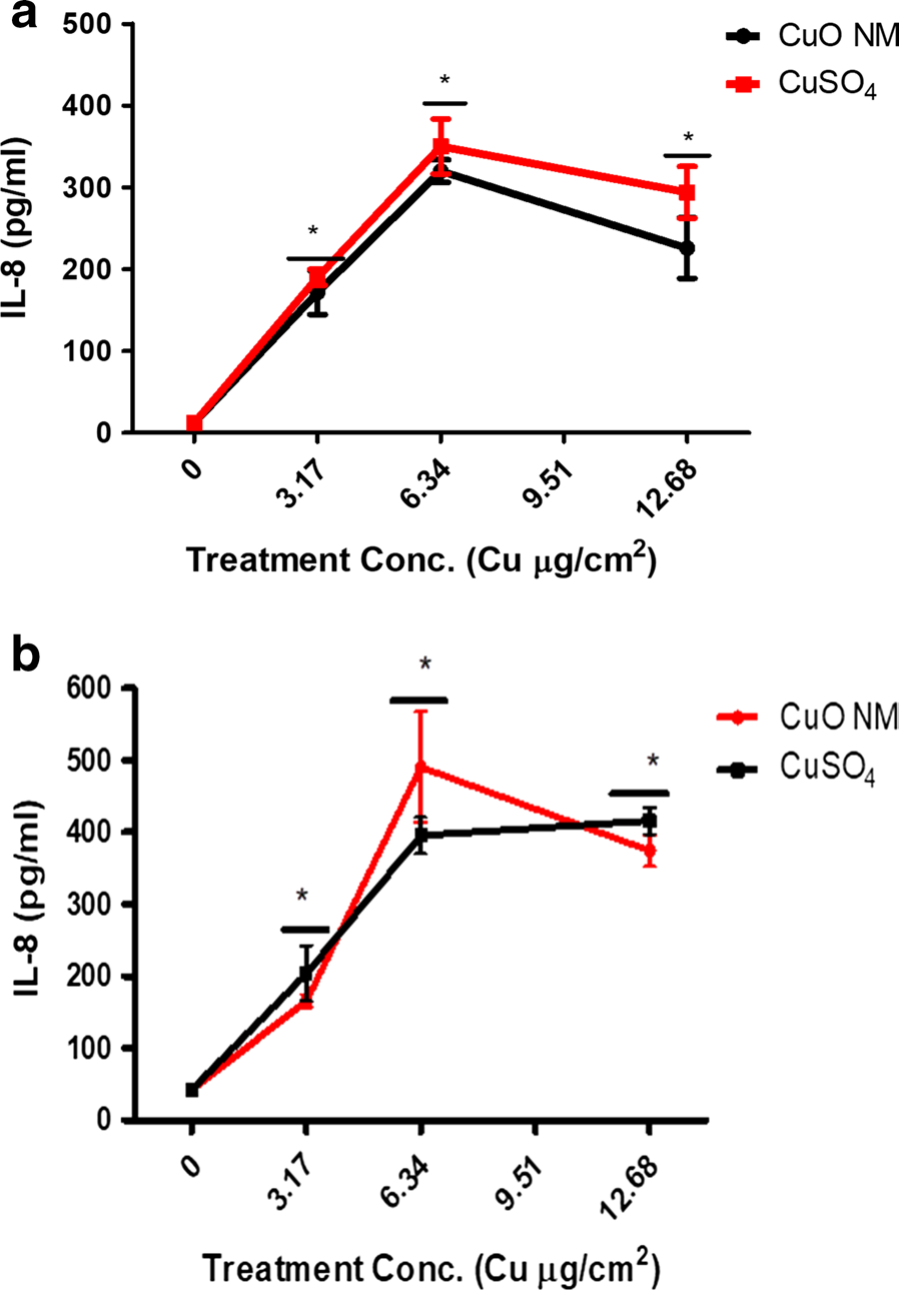
IL-8 production by the Caco-2/HT29-MTX co-culture following exposure to CuO NMs and CuSO_4_. The Caco-2/Raji B (**a**) and Caco-2/HT29-MTX (**b**) co-cultures were exposed to cell culture medium (0), CuO NMs or CuSO_4_ at concentrations of 3.17, 6.34 or 12.68 Cu µg/cm^2^ for 24 h. The level of IL-8 in the cell supernatant was determined using an ELISA. Data are expressed as mean IL-8 concentration (pg/ml) ± SEM (n = 3). Significance at p < 0.05 is indicated by * compared to control

A concentration dependent increase in IL-8 secretion was observed following exposure of the Caco-2/Raji B and Caco-2/HT29-MTX co-cultures to CuO NMs and CuSO_4_ for 24 h (Fig. 9). Significant levels of IL-8 production were observed at all concentrations tested for both treatments, compared to the control (Fig. 9). The Caco-2/HT29-MTX co-culture secreted a higher level of IL-8 compared to the Caco-2/Raji B co-culture. The positive control (200 ng/ml TNF-α) induced secretion of 174.76 ± 41.44 pg/ml of IL-8 by the Caco-2/HT29-MTX co-culture whereas the Caco-2/Raji B co-culture secreted 76.92 ± 13.95 pg/ml. A similar level of IL-8 was produced by cells exposed to CuO NMs and CuSO_4_ at all concentrations for both models. A below detectable level of IL-8 was observed in the supernatant collected from the BL compartment for all treatments (data not shown).

## Discussion

Previously, we demonstrated that CuO NMs and CuSO_4_ caused a concentration dependent decrease in cell viability in undifferentiated cells, and stimulated IL-8 production by undifferentiated and differentiated Caco-2 cells [47]. CuO NMs and CuSO_4_ were also shown to impair the barrier integrity of differentiated Caco-2 cells [47]. Translocation of CuO NMs and CuSO_4_ across the differentiated Caco-2 cell monolayer was also observed [47]. In this study, the impact of CuO NMs and CuSO_4_ on the Caco-2/Raji B and Caco-2/HT29-MTX co-cultures was investigated via assessment of cell viability (nuclei count and light microscopy), cell morphology (light microscopy and SEM), IL-8 production, barrier integrity (SEM, TEER and ZO-1 staining), and Cu translocation (ICP-OES). At the concentrations and time points investigated CuO NMs and CuSO_4_ caused no significant cell death in the Caco-2/Raji B and Caco-2/HT29-MTX co-cultures, as evidenced by no loss of cell number (when assessed via light microscopy and quantification of nuclei number). CuO NMs and CuSO_4_ impaired barrier integrity as shown by a decrease in TEER and reduced tight junction (ZO-1) staining. The compromise in barrier integrity is likely to have promoted the observed concentration and time dependent translocation of Cu particles/ions across the Caco-2/Raji B and Caco-2/HT29-MTX co-culture barriers. CuO NMs and CuSO_4_ also mediated an increase in IL-8 production by both cell models. In general, there was no significant difference between the impact of CuO NMs and CuSO_4_ on the Caco-2/Raji B and Caco-2/HT29-MTX co-cultures for all the endpoints employed, with the exception of IL-8. More specifically, IL-8 production was higher when the Caco-2/HT29-MTX co-culture was exposed to 6.34 Cu µg/cm^2^ CuO NMs compared to the same concentration of CuSO_4_. Overall, the findings suggest that the Caco-2/Raji B co-culture was more sensitive to CuO NMs and CuSO_4_ than the Caco-2 HT29-MTX co-culture for all endpoints investigated.

### Caco-2/Raji B and Caco-2/HT29-MTX co-culture morphology and viability study

In the small intestine, M cells are known to lack some of the functional and morphological characteristics of absorptive cells. For example, they lack microvilli and produce lower levels of hydrolytic enzymes and alkaline phosphatase [18, 31]. Assessment of; cell morphology (using electron microscopy to identify microvilli organisation), TEER, alkaline phosphatase expression/activity/secretion, particle/pathogen transport, and immunostaining and WGA staining have been employed previously to identify the presence of M cells in vitro [17, 24, 26, 29–32, 48]. However, it is noteworthy that published studies have not used a consistent approach to assess the formation of M cells in vitro, with several biochemical and morphological markers employed, in isolation or combination, to date. It is therefore recommended that a combination of approaches are employed in the future to confirm the presence of M cells, and that M cells are identified in a consistent manner across different studies. In this study, FITC labelled WGA staining, SEM and TEM were used to assess the presence of M cells. A higher intensity of WGA staining was observed in the Caco-2/Raji B co-culture compared to the Caco-2/HT29-MTX co-culture, suggesting the successful formation of M cells by Caco-2/Raji B co-culture, which aligns with existing studies [30, 31, 49]. The staining observed in the Caco-2/HT29-MTX co-culture may be because of the presence goblet cells which can also be stained by WGA [50, 51]. However, the pattern of staining is different in the Caco-2/HT29-MTX model which allows M cells to be identified. Of relevance is that it has also been shown some WGA receptors or sialic acid and *N*-acetyl glucosamine are present at a minimal level in epithelial cells [30, 31]. In addition, SEM and TEM demonstrated that in the Caco-2/Raji B co-culture there were areas where microvilli were present in reduced numbers and were more disorganised which suggests the presence of M cells in the co-culture, and aligns with the findings of existing studies [17, 26, 27, 48]. In contrast, the whole surface of the Caco-2/HT29-MTX co-culture was covered in microvilli, and the microvilli were more organised and regular in appearance suggesting a lack of M cells in this model.

The human intestinal epithelium is known to secrete mucus which has important defensive properties. Alcian blue staining of the Caco-2/HT29-MTX co-culture, and SEM demonstrated increased mucus production compared to Caco-2/Raji B co-culture, suggesting that co-culturing of Caco-2 and HT29-MTX cells stimulated mucus secretion, which could withstand several washing procedures. Other researchers have also reported similar results when a ratio of 9:1 of Caco-2 and HT29-MTX cells were co-cultured [30, 37].

TEER measurement is routinely used to study the integrity of differentiated Caco-2 cell models. It has been shown previously that formation of M cells leads to reduced TEER values [17, 21, 48]. Therefore, seeding of Raji B cells into the BL compartment of the Caco-2 cell culture may lead to disruption of the integrity of the monolayer [17, 21, 48]. The TEER value of the Caco-2/Raji B co-culture was lower on the 5th day after Raji B cells were seeded into the BL compartment than that observed for the Caco-2/HT29-MTX co-culture. However, as the TEER value was over 500 Ω cm^2^, this indicates that the barrier was still intact. Assessment of TEER was also measured after exposure of the Caco-2/Raji B and Caco-2/HT29-MTX co-cultures to CuO NMs and CuSO_4_ in order to investigate impacts on barrier integrity. No change in TEER value in control cells was observed after 24 h of both the Caco-2/Raji B and Caco-2/HT29-MTX co-cultures. However, when exposed to CuO NMs and CuSO_4_ the TEER value significantly reduced at 12 and 15 h post exposure to the Caco-2/Raji B and Caco-2/HT29-MTX co-culture respectively, suggesting a disruption in barrier integrity. Previously, decreased TEER values have been reported only at one-time point when investigating impacts of NMs on barrier integrity. For example, Ag NMs caused no reduction in TEER value at 4 h post exposure in an M cell model in vitro [29, 30, 52]. Polystyrene and TiO_2_ NMs have also been reported to induce TEER reduction in the Caco-2/HT29-MTX co-culture at 24 h post exposure [26, 43]. The results presented in this study would suggest that regular, repeated measurements of TEER are made when assessing impacts of NMs on barrier integrity in vitro. Of benefit is that TEER measurements can be made in the same experiment, therefore this approach maximises the amount of data obtained from each experiment.

In addition to TEER measurement, the tight junction protein ZO-1 was stained in the Caco-2/Raji B and Caco-2/HT29-MTX co-cultures after exposure to CuO NMs and CuSO_4_ to investigate impacts on the integrity of the intestinal barrier. Monitoring of tight junction development is frequently investigated by staining the ZO-1 protein in different in vitro models of the intestine including differentiated Caco-2 cells [47, 53, 54] and the Caco-2/HT29-MTX co-culture [26]. Only one published paper has used ZO-1 tight junction staining in Caco-2/HT29-MTX co-cultures to investigate NM toxicity [26], and this study measured responses to TiO_2_ NMs. More specifically, Brun et al. [26] demonstrated that TiO_2_ (12 to 140 nm) reduced the staining intensity of ZO-1 protein in the Caco-2/HT29-MTX co-culture at 24 h post exposure. However, to our knowledge, there is no published paper on ZO-1 staining of CuO NMs exposed Caco-2/Raji B or Caco-2/HT29-MTX co-cultures.

Control Caco-2/Raji B and Caco-2/HT29-MTX co-cultures demonstrated intense ZO-1 staining, indicating the formation of intact tight junctions. When exposed to CuO NMs or CuSO_4_, reduced tight junction staining was observed in the Caco-2/Raji B co-culture. Little impact on ZO-1 staining was observed in the Caco-2/HT29-MTX co-culture. This suggests that CuO NMs and CuSO_4_ may have caused a greater disruption of the integrity of the Caco-2/Raji B barrier compared to the Caco-2/HT29-MTX model, which agrees with the findings from the TEER measurement. The smaller impact of CuO NMs and CuSO_4_ on the Caco-2/HT29-MTX co-culture may be as a result of the presence of mucus in the co-culture, which could limit NM interactions with the cells. Of interest is that the loss of tight junction staining observed in the Caco-2/Raji B co-culture model was less (i.e. there was less damage) when compared to that observed for differentiated Caco-2 cells [47], which suggests that the co-culture models are less sensitive to NM toxicity than a monoculture model.

Electron microscopy has been used for the identification of M cells in published papers, as discussed above. Assessment of cell morphology using electron microscopy can also be used to assess the toxicity of NMs to intestinal cells in vitro. Exposure of CuO NMs to the co-culture caused shortening of microvilli in some cells, compared to the control in both the Caco-2/Raji B and Caco-2/HT29-MTX co-cultures. Although shortening or loss of microvilli has not been reported for the Caco-2/Raji B and Caco-2/HT29-MTX co-cultures previously, exposure of CuO NMs [47] and food grade TiO_2_ [55, 56] have disrupted the microvilli in differentiated Caco-2 cells in existing studies. No impact on the morphology of cells or cell number using SEM, light microscopy and nuclei number count was observed in this study, suggesting there was no cell loss due to treatment of the cells with CuO NMs or CuSO_4_. Cell loss following exposure of undifferentiated Caco-2 cells to CuO NMs or CuSO_4_, studied via light microscopy and nuclei count, have been reported previously, but no loss of differentiated cells were observed [47]. Since CuO NMs interfere with lactate dehydrogenase (LDH) assay [57] and as the transwell plate used are not compatible with the plate reader used to perform biochemical assays which assess cell viability or cytotoxicity (e.g. the alamar assay), light microscopy and nuclei counts were used to assess impact of the test substances on cell viability (via assessment of cell number). We have previously demonstrated that nuclei counts provide a good prediction of the impact of CuO NMs and CuSO_4_ on cell viability (as assessed by the Alamar blue assay in undifferentiated Caco-2 cells) [47]. This suggests that the M cell and mucus secreting intestinal in vitro co-culture models are not as sensitive to CuO NM and CuSO_4_ toxicity as monocultures of undifferentiated Caco-2 cells. Of interest is that oral gavage of 32 mg/kg body weight of CuO NMs to rat for 5 consecutive days before sacrificing at 26th day did not show toxic effect when the histopathology of the stomach, liver and bone marrow, liver enzymes (ALT, AST) and WBC in the blood were assessed [58]. This suggests that CuO NMs investigated in our in vitro study are also relatively non-toxic to the intestine in vivo.

### Translocation of CuO NMs

The M cell in vitro model of the intestinal epithelium has been used previously to assess drug absorption and permeability [30, 52, 59]. Translocation of Ag NMs [29], TiO_2_ [26, 27], aminated and carboxylated polystyrene nanoparticles [17, 23], chitosan-DNA NMs [22], and polystyrene NMs [28] have also been investigated using a Caco-2/Raji B co-culture. Caco-2/HT29-MTX co-cultures have been used to study drug absorption and transportation [30, 60, 61], as well as NM uptake and translocation [42, 43, 45, 46, 62–64]. For example, the Caco-2/HT29-MTX co-culture has been used to investigate the translocation of iron oxide NMs (9–10 nm) coated with either cationic polyvinyl amine (aminoPVA) or anionic oleic acid [64], and neutral, amine and carboxyl-modified polystyrene NMs (50 and 100 nm) [43]. However, none of these studies have used CuO NMs and CuSO_4_ or assessed the level of NMs in the AP and BL compartments, and cell lysate. Instead existing studies have only assessed the level of NMs in the BL compartment when investigating NM translocation across the intestinal barrier. There has not yet been any paper which has directly compared the level of translocation of NMs across Caco-2/Raji and Caco-2/HT29-MTX co-cultures.

A concentration and time dependent translocation of Cu from the AP to BL compartment was observed after exposure of Caco-2/Raji B and Caco-2/HT29-MTX co-cultures to CuO NMs and CuSO_4_. The time and concentration dependent translocation of Cu observed could be because the intestinal barrier was compromised, as demonstrated by the reduction in TEER, and ZO-1 staining intensity. Indeed, a decrease in TEER value has been shown to induce an increase in the translocation of insulin encapsulated chitosan NMs via M cells in vitro [30]. As there was no loss of cell viability when the co-culture models were exposed to CuO NMs and CuSO_4_, cell death is unlikely to be responsible for the decline in barrier integrity observed (e.g. the decrease in TEER value) following exposure of cells to CuO NMs and CuSO_4_. However, as barrier integrity is compromised this is likely to facilitate the translocation of CuO NMs and CuSO_4_ across the intestinal barrier in vitro. Translocation of Cu across the intestinal barrier was higher for the Caco-2/Raji B co-culture than that observed for the Caco-2/HT29-MTX co-culture. CuO NMs and CuSO_4_ caused a greater reduction in TEER in the Caco-2/Raji B co-culture, suggesting that they compromised barrier integrity to a greater extent in this model, which is likely to increase translocation. The increased translocation of Cu may also result from the presence of M cells in the Caco-2/Raji B co-culture, as these cells are responsible for antigen sampling and transport across the intestinal barrier.

Other studies have also investigated NM transport across the intestinal barrier in vitro. A Caco-2/Raji B co-culture exposed to Ag NMs (20 and 30 nm) for 4 h [29] and TiO_2_ for 48 h [27] showed a reduced translocation (< 1.5%) compared to the present study. This could be associated with differences in experimental design (e.g. time point, exposure concentration and the physicochemical properties of NMs (e.g. solubility) investigated). Iron oxide NMs (9–10 nm) coated with either cationic polyvinyl amine (aminoPVA) or anionic oleic acid translocated into the BL compartment (< 2%) 24 h post exposure using the Caco-2/HT29-MTX co-culture [64], which was higher than observation for CuO NMs in this present study. Walczak et al. exposed a Caco-2/HT29-MTX co-culture to neutral, amine and carboxyl-modified polystyrene NMs (50 and 100 nm) for 24 h [43] and observed a translocation of 4.5 and 0.5% for 50 nm and 100 nm neutral polystyrene NMs respectively, suggesting that NM size can influence NM translocation across the intestinal barrier. In addition, up to 6.8% of carboxyl-modified polystyrene NMs were translocated across the intestinal barrier in vivo whereas 1% of the amine modified counterpart was translocated suggesting that negative charge may enhance translocation. In vivo translocation studies demonstrated 0.6% of 48 V-radiolabeled (48 V) TiO_2_ NMs (70 nm) translocated into blood 1 h after intra-oesophageal instillation to rats [65]. In addition, < 1.7% translocation into blood was observed at 1 h and 7 day post administration of polystyrene NMs (50 nm) via oral gavage [66]. Therefore, translocation of NMs in vitro is typically greater than that observed in vivo. However, comparative studies would be required using the same time point and NMs that were used in this study to identify whether in vitro and in vivo models provide similar findings.

Interestingly, the Caco-2/HT29-MTX co-culture demonstrated a higher level of copper concentration in the cell lysate compared to the Caco-2/Raji B co-culture (Fig. 7c). Assessment of Cu concentration in the cell lysates includes quantification of Cu that was internalised by cells, as well as that associated with the cell surface. As the mucus layer remains on the surface of the Caco-2/HT29-MTX co-culture after washing, it is possible that the higher level of Cu in the cell lysate in this model is due to the retention of CuO NMs or Cu ions in the mucus layer. As reviewed by Lock et al. [67], the mucus layer may attract and immobilize particles preventing them from penetrating the cell monolayer. Surprisingly, the translocation of CuO NMs and CuSO_4_ was similar, although the dissolution of CuO NMs was < 50% at 0 h and < 80% 24 h [47]. Since the translocation was investigated via ICP OES, the information about the form in which CuO NMs were translocated (particle or ion) are not available. In the future, imaging of NM uptake using microscopy should be included to elucidate whether the CuO NMs are translocated in their particle or ionic form.

The summation of the detected Cu concentration in the AP, BL and cell lysate did not add up to 100%, suggesting a loss of Cu during the experiment. The inability to recover Cu at 100% may be attributed to increased activation of synthesis of thiol containing proteins including metallothionein proteins, which chelate Cu [68, 69]. It may also be attributed to loss of metal ions as a result of binding of metal ions to materials used for cell culture and for the transport experiment [70, 71] such as cell culture plates and the insert polycarbonate membranes thereby preventing 100% detection. Furthermore, washing of cell monolayers with PBS after removal of AP medium, and before cell digestion may lead to loss of NMs and ions.

The permeability of Cu across the Caco-2/Raji B and Caco-2/HT29-MTX co-cultures was measured by calculating *P*_*app*_. The *P*_*app*_ value was less than 1 × 10^−6^ after 24 h and above 1 × 10^−6^ at 48 h post exposure suggesting that at 24 h post exposed Cu was poorly translocated whereas at 48 h post exposure translocation was greater. Permeability to Cu in the M cell in vitro model was greater than that observed for the Caco-2/HT29-MTX co-culture. This was expected due to the presence of less mucus to act as a barrier, the greater loss of the barrier integrity observed for this model and the role of M cells in antigen sampling. The *P*_*app*_ of this was similar to the *P*_*app*_ of insulin-loaded dextran sulphate/chitosan nanoparticles in vitro M cell models [30] but lower than the *P*_*app*_ of ex vivo study with rat ileum using insulin and thiolated trimethyl chitosan nanoparticles [72–74].

### IL-8 production

CuO NMs and CuSO_4_ stimulated a concentration dependent increase in IL-8 production 24 h post exposure to Caco-2/Raji B and Caco-2/HT29-MTX co-culture models. Similarly, exposure of the Caco-2/HT29-MTX co-culture to Ag NMs (20 nm) and AgNO_3_ demonstrated a concentration dependent increase in IL-8 secretion, although 200 nm Ag NMs had no significant impact, indicating a size dependent effect [38]. However, SWCNT-COOH, MWCNT-COOH and PVP wrapped MWCNT-PVP demonstrated no effect in the production of pro-inflammatory cytokines (TNF-α, IL-1β, IL-6 and IL-8) 8 h post exposure to a Caco-2/HT29-MTX co-culture [75]. Of interest is that both the Caco-2/Raji B and Caco-2/HT29-MTX co-cultures produced less IL-8 compared to the monocultures (undifferentiated and differentiated) of Caco-2 cells when exposed to the same type of CuO NMs and CuSO_4_ [47], suggesting that co-cultures produce less IL-8 in response to CuO NM exposure than more simple models.

The plateaus observed in IL-8 production may be because higher concentrations of CuO NMs and CuSO_4_ (12.68 Cu µg/cm^2^) stimulated the maximum level of IL-8 production from cells before 24 h. In vivo IL-8 is released at the early stage of an infection/injury which helps to recruit antigen-presenting cell (APCs) and neutrophils as part of acute inflammatory response [76]. Therefore, a rapid, and short-lasting production of IL-8 which reaches a plateau is expected. Assessment of cytokine release after treatment of Caco-2/Raji B and Caco-2/HT29-MTX co-culture models with NMs is not frequently performed. No published paper has been identified which has studied cytokine production using the Caco-2/Raji B co-culture exposed to NMs, whereas only two published papers assessed cytokine production (TNF-α, IL-1β, IL-6 and IL-8) by NMs using the Caco-2/HT29-MTX co-culture [38, 75]. Cytokine release from other co-culture models has been performed including BEAS-2B (bronchial epithelial) cells with neutrophils [77], Caco-2 with human macrophages (THP-1) [78], and Caco-2 cells with both THP-1 and human dendritic cells (MUTZ-3) [79], all of which observed increased IL-8 secretion. Therefore, this study is in agreement with others in showing that IL-8 production could be incorporated in future studies when testing NM toxicity using 3D GI tract co-culture models such as the Caco-2/Raji B and Caco-2/HT29-MTX co-culture models. CuO NMs and CuSO_4_ produced a similar level of IL-8 at all concentrations, suggesting particle and ion mediated cytokine induction by CuO NMs.

## Conclusions

The Caco-2/Raji B and Caco-2/HT29-MTX co-culture in vitro models have closer physiological characteristics to intestinal cells in vivo than Caco-2 cell monocultures. This study has demonstrated the incorporation of M cells into an in vitro co-culture of Caco-2 and Raji B cells and mucus into the Caco-2 and HT29-MTX co-culture model. Exposure of the Caco-2/Raji B and Caco-2/HT29-MTX co-culture to CuO NMs and CuSO_4_ compromised barrier integrity (as demonstrated by a reduction in TEER measurement, and a slight decrease in ZO-1 staining), shortened microvilli at some areas, stimulated IL-8 production and a time and concentration dependent Cu translocation. No cell loss was observed in both models at the times and concentration tested following exposure to CuO NMs and CuSO_4_. The impact of CuO NMs and CuSO_4_ on the Caco-2/Raji B and Caco-2/HT29-MTX co-cultures was similar in all endpoints used for this study, suggesting both particle and ion mediated toxicity, as the CuO NMs are not fully soluble at the time points under investigation. CuO NMs and CuSO_4_ mediated a greater impact on the Caco-2/Raji B co-culture compared to Caco-2/HT29-MTX co-culture, which suggests that the presence of mucus may have a protective effect. TEER measurement, and IL-8 secretion, may be suggested as useful endpoints for future screening of (CuO) NM toxicity to in vitro intestinal models as they allow a more rapid and technically easier assessment of toxicity than the other endpoints investigated (e.g. SEM, TEM, ZO-1 staining). ZO-I staining intensity and shortening of microvilli may need to be quantified in the future to determine the level of impact caused by CuO NMs and CuSO_4_ on ZO-I and microvilli of both models. Future work (e.g. https://www.patrols-h2020.eu/) will assess the suitability of these models to screen the toxicity of a wider array of NMs, including repeated exposures over longer durations, as well as using combined versions of the two models (i.e. models with both M cells and mucus secreting cells). The data generated will need comparison to in vivo data, and the ability to reproduce results across different labs will be needed to assess the robustness of each model.

## Materials and methods

### Nanomaterials

CuO NMs were obtained from Plasma Chem, GmbH (Berlin, Germany), in a powdered form as a kind gift from project partners in the FP7 funded project Sustainable Nanotechnologies (SUN). The CuO NMs are a crystalline material with a size range of 15–20 nm (size provided by manufacturer). The Brunauer–Emmett–Teller (BET) method was used to evaluate the specific surface area (47 m^2^/g and a density (6.3 g/cm^3^) (manufacturer data sheet). Previously, Gosens et al. conducted a detailed characterisation of the CuO NMs was performed using transmission electron microscopy (TEM), X-ray diffraction (XRD), and Inductive Coupled Plasma Optical Emission Spectrometry (ICP-OES) [80]. Dissolution studies, size and zeta potential were also previously studied after dispersion in complete cell culture medium [47, 80].

### Nanomaterial preparation

CuO NMs and CuSO_4_ were dispersed following the procedure developed by Jacobsen et al. [81]. Briefly, CuO NMs and CuSO_4_ were dispersed in 2% heat inactivated fetal bovine serum (FBS) in Milli Q de-ionised water and bath sonicated for 16 min without pause. Following the sonication step, all samples were serially diluted in complete DMEM cell culture medium to obtain the required concentration and used immediately.

### Cell culture

The human colon colorectal adenocarcinoma Caco-2 cells and Human Burkitti’s lymphoma; B lymphocyte (Raji B) cells were obtained from the American Type Culture Collection (ATCC) (USA). HT29-MTX cells were obtained from European Collection of Authentic Cell Culture (ECACC) (UK). Caco-2 and HT29-MTX cells were maintained in 4.5 g/l glucose Dulbecco’s modified eagle medium (DMEM) (Sigma) supplemented with 10% heat inactivated FBS (Gibco Life Technologies), 100 U/ml Penicillin/Streptomycin (Gibco Life Technologies), 100 iu/ml NEAA (Gibco Life Technologies), and 2 mM l-glutamine (Gibco Life Technologies), at 37 °C and 5% CO_2_ and 95% humidity. Raji B cells were maintained in Roswell Park Memorial Institute (RPMI) 1640 Medium (Gibco Life Technologies) supplemented with 10% heat inactivated FBS (Gibco Life Technologies), 100 U/ml Penicillin/Streptomycin (Gibco Life Technologies) and at 37 °C, 5% CO_2_ and 95% humidity.

### Culturing of the in vitro intestinal microfold (M) cell and mucus secreting co-culture models

The Caco-2/Raji B co-culture (M cell model) of the gastrointestinal epithelium was cultivated by modifying the protocol previously described by [23, 31, 49]. Briefly, 3.13 × 10^5^ cells/cm^2^ of Caco-2 cells were suspended in 0.5 ml of DMEM and seeded into the AP compartment of 3.0 µm pore polycarbonate transwell inserts in a 12-well plate, with a growth area of 1.12 cm^2^ (Corning) and grown for 15 days at 37 °C, 5% CO_2_ and 95% humidity. The medium on both the AP (0.5 ml) and BL (1.5 ml) compartments was changed every other day. On the 15th day, 5 × 10^5^ cells/ml of Raji B cells were suspended in DMEM (1.5 ml) and seeded into the BL compartment. The co-culture was grown for 5 days under standard incubation conditions and the medium was changed only in the AP compartment every day.

The Caco-2/HT29-MTX co-culture (mucus secreting) model of the gastrointestinal epithelium was cultured by modifying the protocol of [37, 38]. Briefly, 3.13 × 10^5^ cells/cm^2^ of Caco-2 and HT29-MTX cells were seeded onto the AP compartment of 3.0 µm pore polycarbonate transwell inserts in a 12-well plate with growth area of 1.12 cm^2^ (Costar corning, Flintshire, UK) at a ratio of 9:1. The co-culture was maintained in 4.5 g/l glucose DMEM. The cells were cultivated at 37 °C, 5% CO_2_ and 95% humidity for 20–21 days and cell culture medium changed every other day for the first 16 days and then every day until the 21st day and the medium was changed every 2 days. TEER was measured (see below for details) every other day to monitor the development of the intact monolayer in both co-culture models and only co-cultures with TEER values greater than 500 Ω cm^2^ were used for experiments.

### Alcian blue staining

In order to confirm that the Caco-2/HT29-MTX co-culture model produced mucus, Alcian blue staining was used. Alcian blue is a blue dye used to stain mucus due to its specificity to acidic glycoproteins [37]. Briefly, the Caco-2/HT29-MTX co-culture was washed twice with PBS and then fixed with 4% formaldehyde for 25 min at RT. The cells were washed thrice with PBS and stained with 10 mg/ml Alcian blue (in 3% acetic acid) for 30 min at RT. The cells were washed with PBS and the inserts were then carefully excised and mounted onto a microscopic slide. The cells were covered with a glass coverslip, visualised with Axiovert 40 C light microscope (ZEISS, Germany) and the image taken with camera (EOS 60D Canon, Japan).

### Wheat germ agglutinin (WGA) conjugates staining

To confirm the formation of M cells by the Caco-2/Raji B co-culture fluorescein isothiocyanate (FITC) labelled WGA was used due its high affinity for sialic acid and *N*-acetylglucosamine residues present in M cells. It has been shown previously that a high intensity ring-like WGA stain forms around the M cell [31]. Differentiated Caco-2 cells, Caco-2/HT29-MTX and Caco-2/Raji B co-cultures were washed twice with PBS and fixed with 4% formaldehyde for 25 min at RT. The cells were washed three times with PBS and then 500 µl of 5.0 µg/ml WGA FITC conjugate (in PBS) (Sigma Poole, UK) was exposed to cells for 15 min at RT. The cells were then washed two times with PBS. The polycarbonate inserts were carefully excised and mounted with ProLong^®^ Gold antifade reagent with DAPI (Molecular probes, Life Technology, UK) onto a microscope slide and covered with a glass coverslip then sealed with nail polish and allowed to dry at 4 °C for 24 h before visualizing with a Carl Zeiss Axio Scope A 1 Upright Research Microscope (Germany) fixed with a camera (ZEISS Axiocam ERc 5 s). More than 4 fields of view were imaged.

### Impact of CuO NMs and CuSO_4_ on Caco-2/Raji B co-culture barrier integrity

The Caco-2/Raji B and Caco-2/HT29-MTX co-cultures were exposed to cell culture medium (control), 6.34 or 12.68 Cu µg/cm^2^ CuO NMs and CuSO_4_ (500 µl). TEER was measured using an epithelial voltohmmeter EVOM (World precision instrument, Sarasota, USA) every 3 h up to 15 h and then 24 h by putting the short electrode in the AP compartment while the long electrode was placed in the BL compartment touching the cells. The resistance reading in ohms was taken once the reading stabilized. The resistivity was calculated using Eq. 1.

1 $${\text{Resistivity }}\left( {\varOmega \,{\text{cm}}^{2} } \right) = {\text{ohm}}2 - {\text{ohm}}1 \times {\text{A}}$$

where Ohm1 = Resistance of the insert with cell culture medium only, Ohm 2 = Resistance of the insert with cell, A = Surface area of the insert in cm^2^.

TEER are reported as resistivity.

### Immunostaining of Caco-2/Raji B and Caco-2/HT29-MTX co-culture tight junctions

The Caco-2/Raji B and Caco-2/HT29-MTX co-cultures were exposed to cell culture medium (control), CuO NMs or CuSO_4_ (6.34 Cu µg/cm^2^) (500 µl/well) for 24 h at 37 °C and then washed twice with PBS. The cells were fixed with 4% formaldehyde for 25 min at RT and 50 mM ammonium chloride was used to quench excess aldehyde groups at RT for 10 min. The cells were permeabilized with 0.1% triton X100 for 10 min and nonspecific binding was blocked with 10% BSA for 2 h at the RT. Cells were then incubated with 1 µg/ml (diluted in 1% BSA) anti ZO-1 tight junction protein antibody (Abcam, Cambridge, UK) overnight (o/n) at 4 °C. Cells were incubated with Alexa Fluor 488 goat anti-rabbit IgG H&L (Abcam, Cambridge, UK) (secondary antibody), diluted to 4 µg/ml (in 1% BSA) for 1 h. This was followed by counter staining with DAPI (300 nM) for 15 min at RT for nuclei. After each step, cells were washed three times with PBS except after treatment with secondary antibody, which was washed five times. The polycarbonate inserts were carefully excised, mounted with mowiol containing DABCO (antifading agent), covered with a glass coverslip and then sealed with nail polish. The slides were incubated at 4 °C for 24 h before cells were visualized using a Zeiss LSM880 confocal microscope (Germany) and the results analysed using the Zen program and Image J software.

### Nuclei counting (cytotoxicity)

The Caco-2/Raji B and Caco-2/HT29-MTX co-cultures were exposed to cell culture medium (control), CuO NMs or CuSO_4_ (6.34 or 12.68 Cu µg/cm^2^) (500 µl/well) for 24 h at 37 °C, and then were washed twice with PBS and the nuclei stained with DAPI. The polycarbonate inserts were carefully excised and mounted with mowiol containing DABCO (anti fading agent) onto a microscope slide and covered with a glass coverslip, which was then sealed with nail polish and allowed to dry at 4 °C for 24 h before visualizing with a Carl Zeiss Axio Scope A 1 Upright Research Fluorescent Microscope (Germany) fitted with a camera. At least five fields of view (each field of view was 140.80 × 105.60 microns) were imaged for each sample. The results were analysed with Image J software and data are expressed as mean nuclei number ± SEM, and representative images presented.

### Scanning electron microscopy (SEM)

The Caco-2/Raji B and Caco-2/HT29-MTX co-cultures were exposed to cell culture medium (control) or 12.68 Cu µg/cm^2^ of CuO NMs (500 µl/well) for 24 h at 37 °C. After 24 h, the inserts were washed with PBS twice, fixed with 5% glutaraldehyde (in 0.1 M sodium cacodylate) for 2 h at 4 °C. The cells were washed three times with 0.1 M sodium cacodylate followed by dehydration in graded ethanol (25, 50, 70, 80 and 90%) for 10 min in each ethanol grade at RT. The cells were further dehydrated in 100% ethanol three times for 15 min each then, submerged in 2:1 fresh solution of hexamethyldisilazane (Sigma, Poole):100% ethanol. The glass coverslips containing the cells were dried in 100% hexamethyldisilazane (Sigma, Poole) and mounted on SEM specimen stubs (Aluminium, 12.5 mm diameter, 3.2 × 6 mm pin Agar Scientific UK). The polycarbonate membranes were carefully excised and mounted on SEM specimen stubs (Aluminium, 12.5 mm diameter, 3.2 × 6 mm pin Agar Scientific UK). Then, specimens were coated with gold and examined with SEM. More than 5 views were imaged, and representative images presented.

### Romanowsky staining (assessment of cytotoxicity and cell morphology)

The Caco-2/Raji B and Caco-2/HT29-MTX co-cultures were exposed to cell culture medium (control), 6.34 and 12.68 Cu µg/cm^2^ of CuO NMs or CuSO_4_ (500 µl/well) at 37 °C. After 24 h, the cells were stained with Rapid Romanowsky stain (TCS Biosciences, England). Briefly, differentiated Caco-2 cells were fixed by rinsing the insert 15 times in methanol. To stain the cells the inserts were then rinsed with eosin Y solution 15 times and then methylene blue solution 15 times. The inserts were thoroughly washed in distilled water and air-dried. The polycarbonate inserts were carefully excised, then mounted with DPX (Sigma, Poole UK) and covered with a glass coverslip. The specimens were viewed and imaged using a light microscope-Zeiss fluorescent microscope, Carl Zeiss Axio Scope A 1 Upright Research Microscope (Germany) fitted with camera (ZEISS Axiocam ERc 5 s) (magnification 40×).

### IL-8 production

The Caco-2/Raji B and Caco-2/HT29-MTX co-cultures were treated with cell culture medium (control), 200 ng of TNF-α (positive control), 3.17, 6.34 or 12.68 Cu µg/cm^2^ of CuO NMs and CuSO_4_ (500 µl/well), and IL-8 secretion was then assessed using Enzyme-linked Immunosorbent Assay (ELISA) (R&D System, Inc., Minneapolis, MN USA) following the manufacturer’s protocol. The absorbance of human IL-8 production was measured using a SpectraMax M5 microplate reader (California USA) at wavelength 450 nm and the concentration in pg/ml was obtained from the standard curve. Data are expressed as mean IL-8 concentration (pg/ml).

### Assessment of Cu ion concentration

The Caco-2/Raji B and Caco-2/HT29-MTX co-cultures were exposed to complete cell culture medium (control), 3.17, 6.34 and 12.48 Cu µg/cm^2^ of CuO NMs and CuSO_4_ (500 µl/well) for 24 or 48 h at 37 °C, 5% CO_2_ and 95% humidity. This was followed by removal of the cell culture medium from the AP and BL compartments. The medium from the AP (300 µl) and BL (900 µl) compartments were digested with 5 ml of 4% HNO_3_ (Sigma), filtered with Puradisc 25 mm 0.2 µm PES filter media (Whatman). The volume was made up to 10 ml with Milli Q deionised H_2_O to obtain final acidic concentration of 2% HNO_3_.

The cells were also digested following the method previously described [82]. Briefly, to detach the cells, 25 mM trypsin EDTA (100 µl) was added into the apical compartment of the transwell plate and incubated for 5 min at 37 °C, 5% CO_2_ and 95% humidity. The cells were then digested by adding 1 ml of 20% HNO_3_ followed by shaking with a rotatory shaker, PMS-1000, Grant-bio (Cambridge UK) at high speed for 4 h, at RT. The cell suspension was then diluted with Milli Q H_2_O to get an acidic concentration of 2% HNO_3_, and then filtered with Puradisc 25 mm 0.2 µm PES filter media (Whatman). The copper ion content in the acidic extracts of medium (from AP and BL compartment) and cell were analysed by Dr. Lorna Eades (University of Edinburgh) using Inductive Coupled Plasma Optical Emission Spectrometry (ICP-OES) (Perkin Elmer Optima 5300 DV USA). Radio frequency (RF) forward power of 1400 W, with argon gas flows of 15, 0.2 and 0.75 l/min for plasma, auxiliary, and nebuliser flows was employed respectively. Data are expressed as % of the exposed concentration.

Apparent permeability coefficients (*P*_*app*_) of Cu were calculated using Eq. 2 [17].

2 $${\text{Papp }}\left( {{\text{cm}}/{\text{s}}} \right) = \frac{{\Delta {\text{Q}}}}{{\Delta {\text{t}}}} \times \frac{1}{\text{A}} \times {\text{Co}}$$

where ∆Q/∆t is the amount of Cu transported into the BL compartment per unit time (t), A is the surface area of the insert (Caco-2 cell monolayer) and Co is the initial concentration of the substance in the donor (AP) compartment.

### Data analysis

Each experiment was repeated at least three times (on different days) and all data generated from these experiments are expressed as the mean ± Standard error of the mean (SEM). The figures were generated using Graph Pad Prism. After checking normality of the data, a one-way analysis of variance (ANOVA) with a Tukeys multiple comparison was employed to investigate statistical significance using Minitab 17 software. The microscope images were analysed with image J software.

## Additional files

**Additional file 1.** TEER value of the Caco-2/HT29-MTX co-culture over 21 days. Caco-2/HT29-MTX cells were grown in transwell plates, and TEER measurement made at regular intervals to monitor cell differentiation. Data are expressed as mean TEER value ± SEM (n = 3).

**Additional file 2.** TEER value of the Caco-2/Raji B co-culture over 20 days. A Caco-2/Raji B co-culture was grown in transwell plates, and TEER measurements made at regular intervals to monitor cell differentiation. Data are expressed as mean TEER value ± SEM (n = 3).

## Data Availability

Data supporting the findings are presented within the manuscript and additional files. Raw data files are available on request to the corresponding author.

## Abbreviations

CuO NMs: copper oxide nanomaterials
CuSO_4_: copper sulphate salt
TEER: transepithelial electrical potential
FITC: fluorescein isothiocyanate
ANOVA: analysis of variance
EDTA: ethylenediaminetetraacetic acid
PBS: phosphate buffered saline
FBS: fetal bovine serum
IL-8: interleukin-8
DABCO: 1,4-diazabicyclo[2.2.2]octane
WGA: wheat germ agglutinin
TEM: transmission electron microscopy
SEM: scanning electron microscopy
ICP-OES: Inductive Coupled Plasma Optical Emission Spectrometry
*P*_*app*_: apparent permeability coefficients
ELISA: enzyme-linked immuno-sorbent assay
