# Supplementary material for: Using 3D gastrointestinal tract in vitro models with microfold cells and mucus secreting ability to assess the hazard of copper oxide nanomaterials
Source: J Nanobiotechnology. 2019 May 21;17:70. doi: 10.1186/s12951-019-0503-1 (PMC6530093; doi:10.1186/s12951-019-0503-1)
Supplement: Supplementary file 1 — Additional file 1. TEER value of the Caco-2/HT29-MTX co-culture over 21 days. Caco-2/HT29-MTX cells were grown in transwell plates, and TEER measurement made at regular intervals to monitor cell differentiation. Data are expressed as mean TEER value ± SEM (n = 3). [file 12951_2019_503_MOESM1_ESM.doc]

**Additional file 1**: TEER value of the Caco-2/HT29-MTX co-culture over 21 days.

Caco-2/HT29-MTX cells were grown in transwell plates, and TEER measurement made at regular intervals to monitor cell differentiation. Data are expressed as mean TEER value ± SEM (n = 3).
