# Supplementary material for: Using 3D gastrointestinal tract in vitro models with microfold cells and mucus secreting ability to assess the hazard of copper oxide nanomaterials
Source: J Nanobiotechnology. 2019 May 21;17:70. doi: 10.1186/s12951-019-0503-1 (PMC6530093; doi:10.1186/s12951-019-0503-1)
Supplement: Supplementary file 2 — Additional file 2. TEER value of the Caco-2/Raji B co-culture over 20 days. A Caco-2/Raji B co-culture was grown in transwell plates, and TEER measurements made at regular intervals to monitor cell differentiation. Data are expressed as mean TEER value ± SEM (n = 3). [file 12951_2019_503_MOESM2_ESM.doc]

**Additional file 2**: TEER value of the Caco-2/Raji B co-culture over 20 days.

A Caco-2/Raji B co-culture was grown in transwell plates, and TEER measurements made at regular intervals to monitor cell differentiation. Data are expressed as mean TEER value ± SEM (n = 3).
